# Supplementary material for: How GBS Got Its Hump: Genomic Analysis of Group B Streptococcus from Camels Identifies Host Restriction as well as Mobile Genetic Elements Shared across Hosts and Pathogens
Source: Pathogens. 2022 Sep 8;11(9):1025. doi: 10.3390/pathogens11091025 (PMC9504112; doi:10.3390/pathogens11091025)
Supplement: Supplementary file 1 [file pathogens-11-01025-s001.zip › Supplementary File S4.pdf]

Supplementary Materials: How GBS Got its Hump: Genomic Analysis of Group B *Streptococcus* from Camels Identifies Host Restriction as well as Mobile Genetic Elements Shared Across Hosts and Pathogens

Chiara Crestani, Dinah Seligsohn, Taya L. Forde and Ruth N. Zadoks

1. Figures

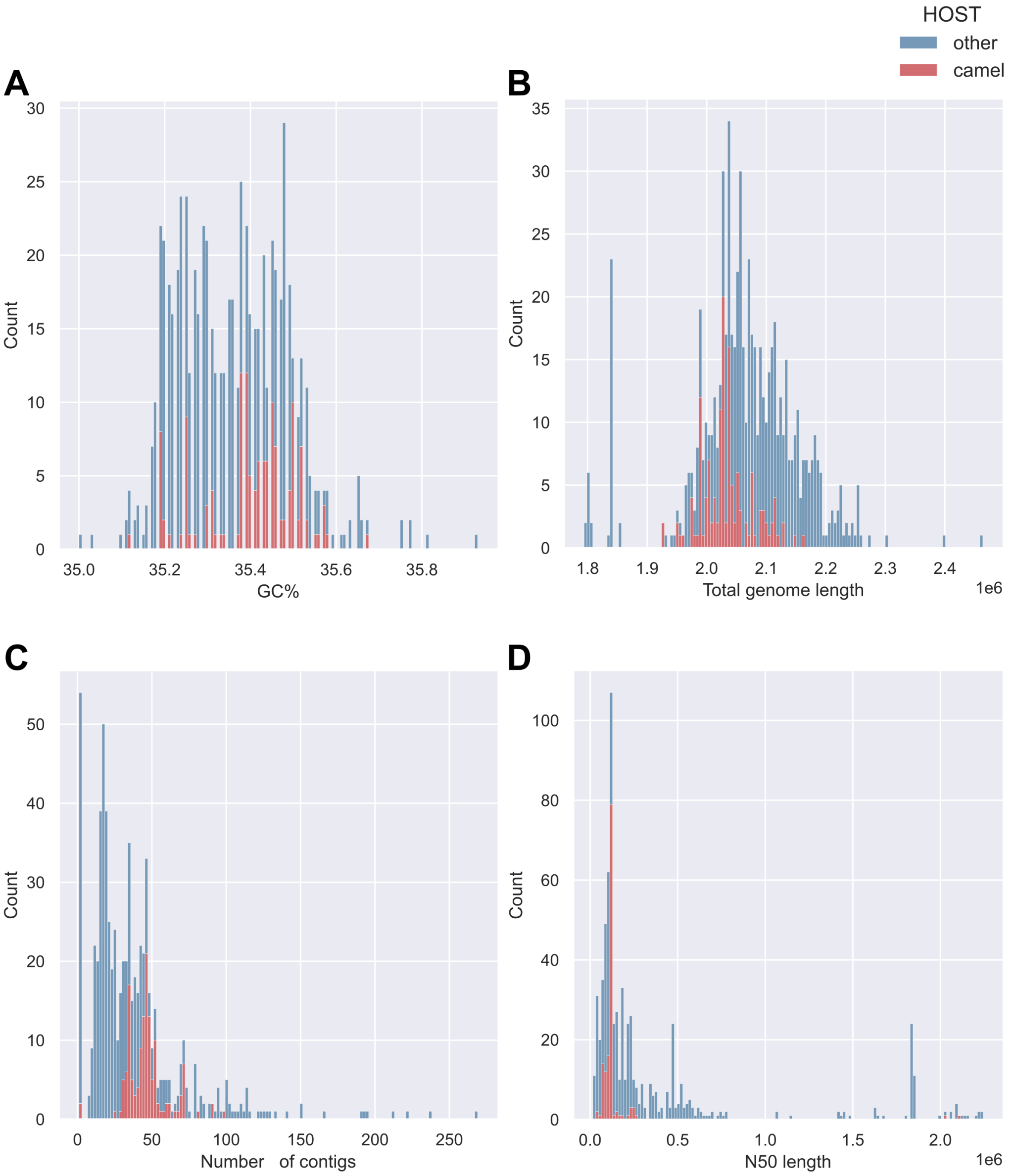

Figure S1. Frequency distribution plots of quality parameters evaluated with QUAST for 680 Group B *Streptococcus* isolates from humans and animals: A) GC content (%); B) total genome length (in base pairs); C) Number of contigs; D) N50 length (in base pairs). Coloured bars show the number of genomes (y-axis) and the value (x-axis) for genome assemblies from camel isolates (red) and from other host species (blue) included in this study.

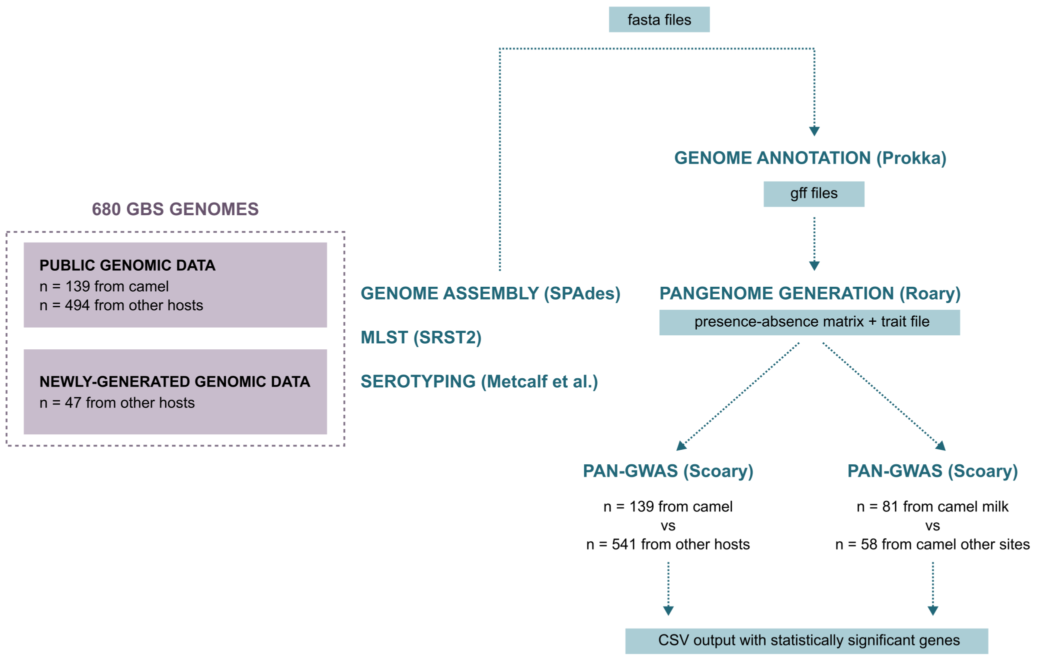

Figure S2. A flow chart of the methods used for GWAS analyses in this paper.

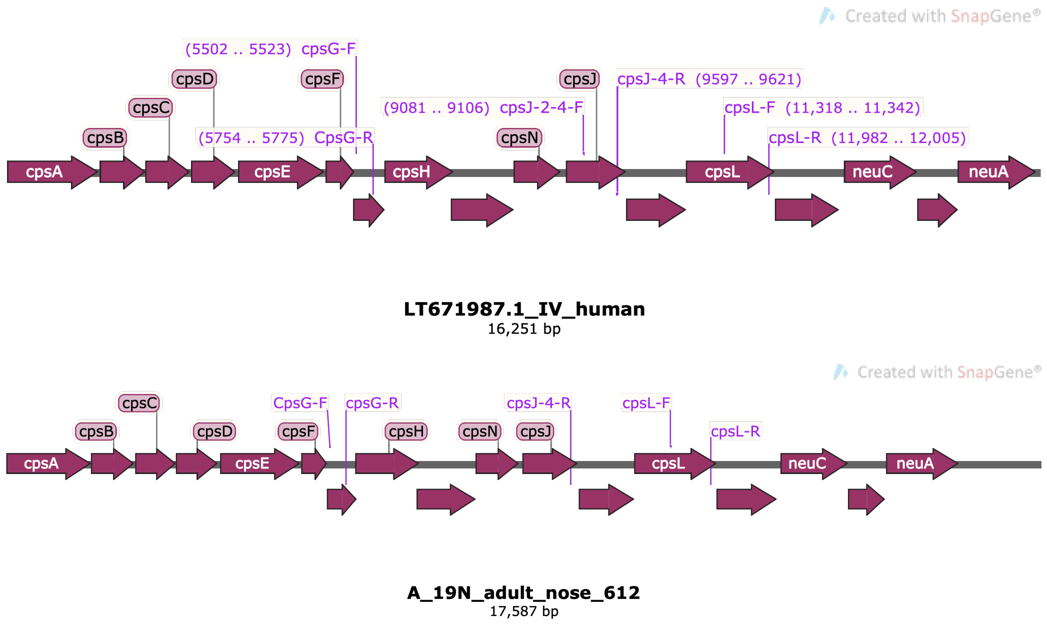

Figure S3. Comparison of binding sites for primers used for the amplification of Group B *Streptococcus* capsular operon genes *cpsG*, *cpsJ* and *cpsL* from Imperi et al. (2010). In this multiplex PCR method, the *cpsJ* band is used to distinguish capsular type Ia (amplicons for *cpsG* and *cpsL*) from type IV (amplicons for *cpsG*, *cpsJ* and *cpsL*). The human reference sequence for capsular type IV (top) shows binding sites for primer pairs for each of the three genes. The example for type IV in camels (bottom) shows no binding for the forward primer for *cpsJ* amplification, but has binding sites for forward and reverse primers of *cpsL* and *cpsG*.

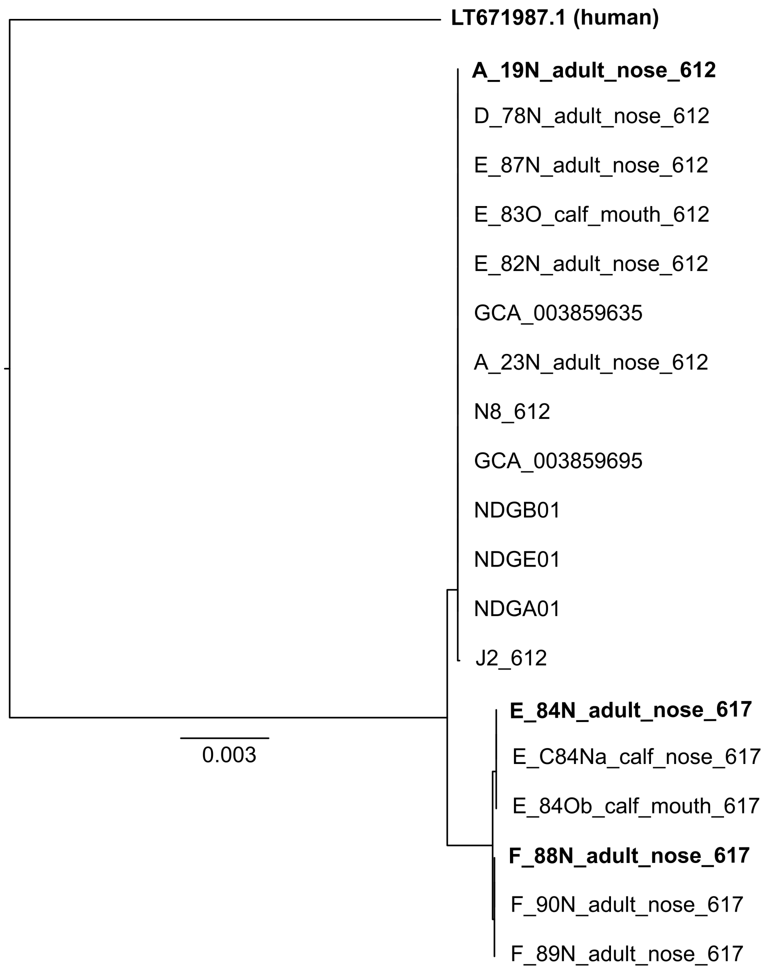

Figure S4. Maximum-likelihood phylogenetic tree of capsular type IV loci extracted from group B *Streptococcus* genomes from camels and a human reference isolate. Genomes in boldface correspond to genomes in Fig. 2. Tree was rooted at midpoint.

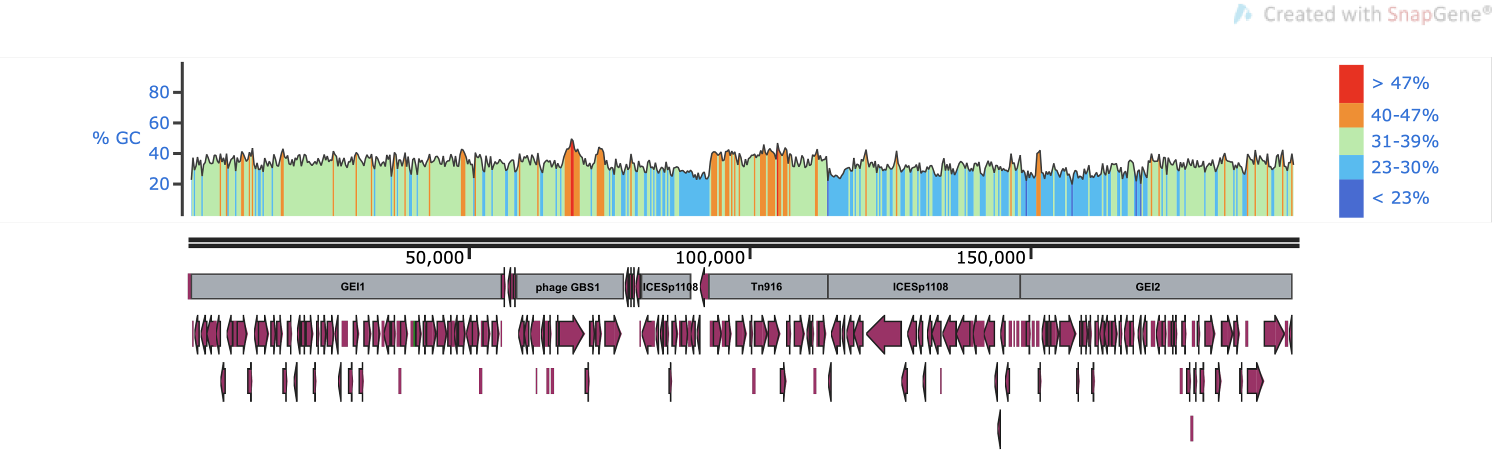

Figure S5. Map showing the camel-associated genomic islands (GEI1 and GEI2) of Group B *Streptococcus*, and the region between the two, which contains partial prophage GBS1, ICE*Sp*1108 (in two segments) and Tn*916*. The graph above shows the GC content (%).

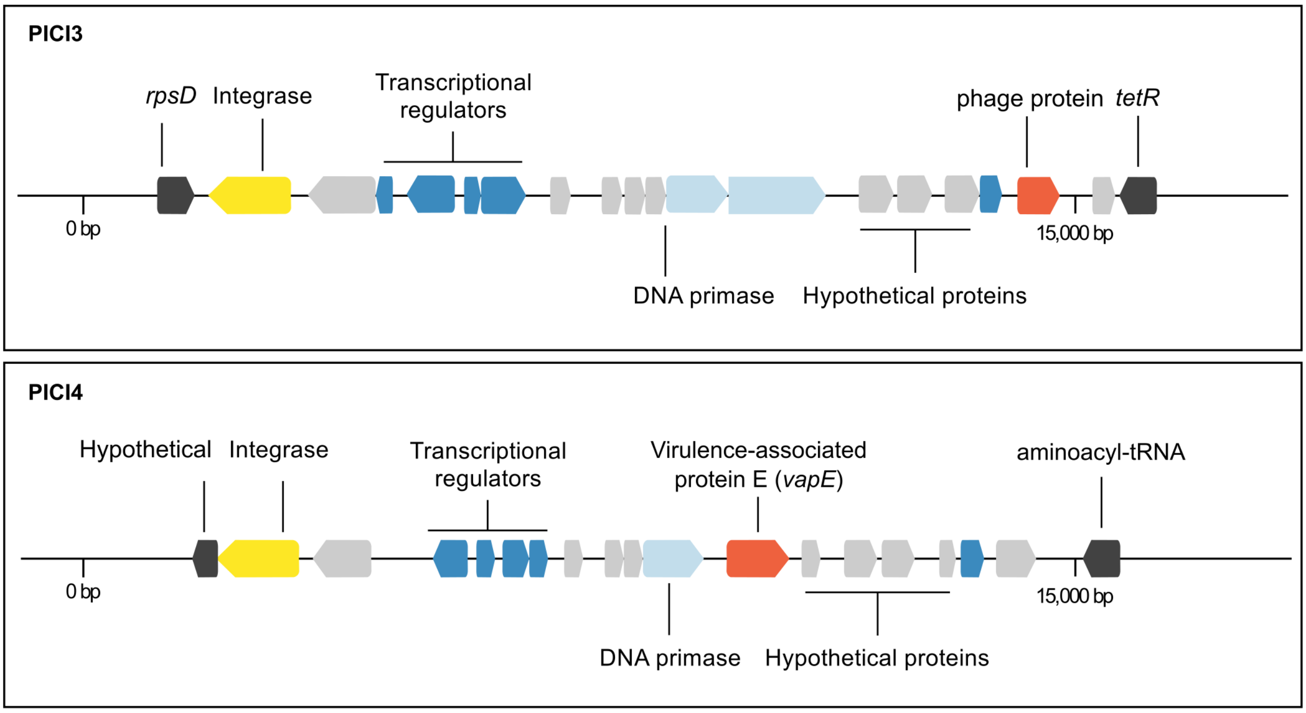

Figure S6. Annotated maps of genes in phage-inducible chromosomal island (PICI) 3 (isolate 84Ob) and PICI4 (isolate E7) of Group B *Streptococcus* from camels. The integration site of PICI3 is the same as that of PICI1 and PICI2 (Crestani et al., 2020), the *rpsD* gene, whereas for PICI4 it is next to a hypothetical gene. Genes are colour-coded based on function (black: chromosomal genes; yellow: site-specific integrase; dark blue: lysogeny genes; light blue: replication genes; light grey: hypothetical; red: other genes).

2. Phage-inducible chromosomal islands integrase types in Group B *Streptococcus*

PICI3*Int*

MKITEIKKKNGATVYRASIYLGVDQVTGKKVKTKVTGRTQKEVKQKANQEKIAFQK
DGCTRFKATSIASYQELSNLWWESYKHTVKPNTQDNVKKLLDNHVLPLFGGYKLDK
LTTPLIQSIVNKLADKTNKGEPGAYLHYDKIHALNKRILQYGVTMQAIPANPARDVV
LPRNTQKAKRKKVKHFENQDLKKFLDYLGGLDLAKYRNLYEITLYKFLLATGCRINE
ALALSWSDIDLENATISITKTLNHLGQINSPKSKASYRDIDIDQGTVSMLKTYQLRQIQ
EAWKLGQTETVVFSDFIHDYPSNKTLGTRLKTRFKRAGVPNIGFHGFRHTHASLLLN
SGIPYKELQHRLGHSTLSMTMDIYSHLSKENAKKAVSFYETALK

PICI4*Int*

MERFMIMKITEVKKKDGTVIYRASIYLGIDQVTGKKAKTSVTGRTRKEVKQKARHAQ
DEFISNGYTVTKVVPIKNYQELAELWLENYQLTVKPQTFIATKRMLYNHLIPIFGTMK
VDKLTVSYIQRFINDLSNQLVHYGVVHSINRRVLQYGVSLQLLPFNPARDVMLPKVP
KKENKAIKFIAPEDLKALMAYMEKLANKKFSYFFDYVLYSVLLATGCRFGEVVALEW
SDIDLENGTISITKNYSRLLKLIGTPKSKAGVRVISIDKKTINLLRLYKNRQRQLFIETGT
RVSAVVFSTPLKEYQNMATRQESLDRRITEVGIPRFTFHAFRHTHASLLLNAGISYKEL
QYRLGHATLAMTMDIYGHLSMDKEKEAVSYFEKAIN
